# Prevalence and Profiles of Antibiotic Resistance Genes *mph*(A) and *qnrB* in Extended-Spectrum Beta-Lactamase (ESBL)-Producing *Escherichia coli* Isolated from Dairy Calf Feces

**DOI:** 10.3390/microorganisms10020411

**Published:** 2022-02-10

**Authors:** Alexis M. Carey, Sarah F. Capik, Sarah Giebel, Colette Nickodem, Juan M. Piñeiro, Harvey Morgan Scott, Javier Vinasco, Keri N. Norman

**Affiliations:** 1 Department of Veterinary Integrative Biosciences, College of Veterinary Medicine and Biomedical Sciences, Texas A&M University, College Station, TX 77843, USA; amcarey@tamu.edu (A.M.C.); cnickodem@tamu.edu (C.N.); 2 Department of Veterinary Pathobiology, College of Veterinary Medicine and Biomedical Sciences, Texas A&M University, College Station, TX 77843, USA; Sarah.Capik@ag.tamu.edu (S.F.C.); hmscott@cvm.tamu.edu (H.M.S.); JVinasco-Torres@cvm.tamu.edu (J.V.); 3 Texas A&M AgriLife Research, Texas A&M University System, Amarillo, TX 70106, USA; 4 Giebel Veterinary Services, Canyon, TX 79015, USA; sgiebel@gmail.com; 5 Texas A&M AgriLife Extension Service, Department of Animal Science, Texas A&M University System, Amarillo, TX 79106, USA; Juan.Pineiro@ag.tamu.edu

**Keywords:** *Escherichia coli*, resistance, dairy, calves

## Abstract

The use of antibiotics to treat dairy calves may result in multidrug-resistant extended-spectrum beta-lactamase (ESBL)-producing *Escherichia coli*. This study investigated fluoroquinolone and macrolide resistance genes among ESBL-producing *E. coli* isolated from dairy calves. Fresh fecal samples from 147 dairy calves across three age groups were enriched to select for ESBL-producing *E. coli.* Plasmid-mediated fluoroquinolone (*qnrB*), macrolide (*mph*(A)), and beta-lactam (*bla*_CTX-M_ groups 1 and 9) resistance genes were identified by PCR and gel electrophoresis in ESBL-producing *E. coli*. Beta-lactamase variants and antibiotic resistance genes were characterized for eight isolates by whole-genome sequencing. Seventy-one (48.3%) samples were positive for ESBL-producing *E. coli*, with 159 (70.4%) isolates identified as *bla*_CTX-M_ variant group 1 and 67 (29.6%) isolates as *bla*_CTX-M_ variant group 9. Resistance gene *mph*(A) was more commonly associated with *bla*_CTX-M_ variant group 1, while resistance gene *qnrB* was more commonly associated with variant group 9. *E. coli* growth was quantified on antibiotic media for 30 samples: 10 from each age group. Significantly higher quantities of ceftriaxone-resistant *E. coli* were present in the youngest calves. Results indicate the dominant *bla*_CTX-M_ groups present in ESBL-producing *E. coli* may be associated with additional *qnrB* or *mph*(A) resistance genes and ESBL-producing *E. coli* is found in higher abundance in younger calves.

## 1. Introduction

Antibiotics are crucial for treating bacterial infections in humans and animals; however, drug-resistant pathogens can limit treatment options available. Many antibiotic classes remain critically important to human medicine, such as third-generation and higher cephalosporins, fluoroquinolones, and macrolides with drug resistance to any of these classes considered a serious threat to human health [1,2]. Antibiotic stewardship in food animal production has limited the use of antibiotics to U.S. FDA-approved therapeutics with defined extralabel usage guidelines to reduce the risk of antibiotic resistance and potential cross-over into human pathogens; however, resistance remains common in food animals, posing a considerable public health concern [3,4,5]. Monitoring foodborne pathogens for existing and emerging antibiotic resistance profiles is essential to identifying potential public health threats.

*Escherichia coli* generally are non-pathogenic and commensal organisms in humans and animals, but their potential to serve as a reservoir for antibiotic resistance poses additional risks to public health through environmental cross-contamination of pathogenic *E. coli* or dissemination of multidrug resistance to other enteric pathogens. In agriculture, the ease of culturing allows *E. coli* to be monitored as an indicator organism for existing and emerging drug-resistant profiles circulating through the fecal-environmental-oral continuum [6]. Considerable in vitro evidence exists for the potential transfer of antibiotic resistance via plasmids between *E. coli* and other enteric pathogens such as *Salmonella* [7]. While little in the way of in vivo evidence has directly captured the active transfer of antibiotic resistance elements from *E. coli* to other gram-negative bacteria, fecal bacteria have great potential to be a source of exposure for human intestinal microflora through consumption of contaminated food products [3,8]. Extended-spectrum beta-lactamase (ESBL)-producing *E. coli* is listed by the U.S. Centers for Disease Control and Prevention (CDC) as a serious threat to human health, and the harboring of additional resistance to other antibiotic classes are of growing concern [1].

Antibiotic co-resistance to cephalosporins, fluoroquinolones, and macrolides has been observed in bacteria, most often *E. coli*, isolated from dairy calves, adult dairy cattle, as well as environmental systems [9,10,11]. The use of antibiotics provides exposure for the potential selection of resistance in a population. For example, the emergence and expansion of ESBL-producing *E. coli* in food animal production has been closely linked to the approval of ceftiofur, a third-generation cephalosporin, that is used to treat various illnesses in dairy calves, as well as other beef and adult dairy cattle [12,13]. Additionally, macrolide antibiotics administered for the treatment of bovine respiratory disease in dairy calves may provide the selective pressures sufficient for resistance to arise [13,14]. The potential transfer of macrolide resistance genes acquired by commensal *E. coli* to other enteric pathogens is problematic as macrolides are the primary treatment in humans for other enteric pathogens, such as those responsible for shigellosis or salmonellosis [15,16]. Similar to macrolide resistance, plasmid-mediated quinolone resistance (PMQR) is especially concerning because resistance can be horizontally transferred increasing the potential for faster expansion of fluoroquinolone resistance [17,18]. A higher generation (third or fourth) of cephalosporin resistance accompanied by macrolide and fluroquinolone resistance is of great concern to human health, as these are antibiotics primarily used to treat human bacterial infections.

Young dairy calves encounter stressors such as maternal separation, transport to rearing farms, or changes in their environment that increase the risk of infection and subsequent need for treatment with antibiotics [19]. Cephalosporins, fluoroquinolones, and macrolides are commonly used to treat disease in dairy calves [20]. Fluoroquinolone and macrolide therapies are only available for use in dairy calves and non-lactating cattle under 20 months of age [21,22]. Cephalosporins, such as Ceftiofur Crystalline Free Acid, are available for use in adult dairy cattle with the restriction of a 13-day withholding period before slaughter and following labeled use no milk withholding is required [23]. In pre- and weaned calves, a relationship between calf age and prevalence of antibiotic-resistant *E. coli* has been observed, with younger calves harboring higher quantities of antibiotic-resistant *E. coli* [8]. Other research has also suggested young calves are a reservoir for antibiotic-resistant *E. coli*, either as a pathogen host or by the secretion of antibiotic residues into the environment, providing adequate selective pressures for resistance to arise outside the host [24,25]. In addition to antibiotic-resistant *E. coli* observations in young calves, previous studies have also identified *E. coli* from adult dairy cattle containing multidrug resistance to antibiotics, including those illegal to administer in adults over 20 months of age [13]. The selective pressures responsible for multidrug-resistant ESBL-producing *E. coli* in dairy calves and cattle remain unclear, but it seems likely that calf age and antibiotic use are important factors to consider.

The use of cephalosporins, fluoroquinolones, and macrolides in dairy calves provides the potential for co-selection of multidrug-resistant ESBL-producing *E. coli*. Determining potential selection factors for multidrug-resistant ESBL-producing *E. coli* in dairy calves and cattle will be valuable due to the highest-priority and critical importance of these antibiotics to human health and the potential for cross-over of antibiotic resistance into human pathogens and the environment. This study was designed to identify ESBL-producing *E. coli* that also harbor the antibiotic resistance genes *mph*(A) and *qnrB* in fecal samples collected from hutch, weaned, and yearling dairy calves.

## 2. Materials and Methods

### 2.1. Sample Collection

A single collection of fresh fecal samples was collected per rectum from 150 dairy calves sampled conveniently from three areas, corresponding to three age groups (50 from each hutch individual housing, weaned group housing, and yearling group housing) from a calf-raising farm in the southern United States during the month of February 2020. Fecal samples were transported at approximately 4 °C on ice to the laboratory. Upon arrival to the laboratory, glycerol stocks consisting of an equal amount of fecal sample and 50% glycerol were made and stored at −80 °C for further culturing and characterization.

### 2.2. Calf Management and Treatment Protocols

Calves arrived at the calf-raising farm 6–36 h after birth from their farm of origin, apart from a few dairy farms sending calves at later time points. All calves were administered prophylactic penicillin upon arrival to the calf-raising farm, as well as given milk replacer supplemented with neomycin for 14-days. Additionally, weaned and yearling heifers received prophylactic oxytetracycline when moved from individual to group housing. As a result, all hutch calves had been treated with aminoglycoside and penicillin antibiotics at least once and all weaned and yearling calves had been treated with aminoglycoside, penicillin, and tetracycline antibiotics at least once prior to fecal sample collection. All other antibiotic classes administered were for the treatment of a health event only with no inclusion criteria for sampling post antibiotic treatment.

Pen movements of calves occur at multiple points, according to calf age or farm management needs. Calves were weaned from milk at 60 days but remained in poly hutches until 90 days, from which they were moved to group pens of 20 calves per pen. At approximately 120 days, calves were then moved to larger pens of approximately 100 calves per pen. Heifers were then moved again into the breeding pens at 395 days, which house 100–120 heifers per pen. While calf management states calves were weaned at 60 days, all calves in hutches received milk regardless of age and were considered pre-weaned.

Pre-weaning, calves were observed by farm personnel for signs of illness at each milk feeding. Weaned calves were observed once a day by farm personnel for signs of illness. In addition to prophylactic treatments, records of health events requiring treatment defined by the calf-raising farm (which included the quantity and type of antibiotic administered) were collected for all 150 calves. The health event definitions are as follows; treatment for scours events occurred for decreased appetite and loose manure; respiratory treatment for pneumonia in the hutches with decreased appetite and increased respiratory effort and pneumonia in group pens with elevated rectal temperature (>103 °F) and depression and increased respiratory effort; eye treatment occurred for pinkeye characterized by tearing, squinting, with presence or absence of an ulcer. Antibiotics administered for health events requiring treatment were obtained from computer records and exported to an Excel spreadsheet (Microsoft Corp., Redmond, WA, USA).

### 2.3. Microbiological Processing for ESBL-Producing E. coli

Of the 150 samples collected, 149 were screened for ESBL-producing *E. coli*; 1 sample in the weaned group was missing from saved glycerol stocks and not processed. Enrichment for ESBL-producing *E. coli* began with the dilution of 1 g of fecal glycerol stock into 9 mL of 1× Gibco^®^ phosphate buffered saline (PBS) (Thermo Fisher Scientific, Gaithersburg, MD, USA), this resulted in a 1:20 dilution factor. Next, 1 mL of the PBS solution was transferred into 9 mL of MacConkey broth (BD Difco™, Franklin Lakes, NJ, USA) supplemented with 2 µg/mL ceftriaxone (Sigma-Aldrich, St. Louis, MO, USA). Enrichment of samples in MacConkey broth with ceftriaxone consisted of two incubation time points at 3 and 6 h. An additional 9-h time point was completed for the yearling group, as this was the first group processed. Enrichments at 3 and 6 h provided the best time points for measuring ESBL growth while maintaining isolated colonies; thus, only two incubation time points were completed for hutch and weaned groups. After 3, 6, or 9 h of total enrichment, 50 µL of MacConkey broth with ceftriaxone was spiral plated to CHROM-ESBL agar (CHROMAgar, Springfield, NJ, USA) using the Eddy Jet 2^®^ system (Neutec Group Inc., Farmingdale, NY, USA). CHROM-ESBL agar was incubated for 18–24 h at 37 °C and checked for pink, lactose fermenting colonies to evaluate positive *E. coli* growth. CHROM-ESBL agar plates were then replica plated to MacConkey agar with 16 µg/mL cefepime (Sigma-Aldrich, St. Louis, MO, USA) using a velvet patch and incubated for 18–24 h at 37 °C. MacConkey agar with cefepime further selected for ESBL-producing isolates likely to contain *bla*_CTX-M_ resistance gene, as cefepime is a fourth-generation cephalosporin only allowing for ESBL-producing bacterial growth. After incubation, three or four lactose fermenting, pink, isolated colonies were selected from MacConkey agar with cefepime and isolated on Tryptic Soy Agar (TSA) with 5% Sheep Blood (BD Difco™, Franklin Lakes, NJ, USA) and incubated for 18–24 h at 37 °C. All presumptive ESBL-producing *E. coli* isolates were preserved on CryoCare beads (Scientific Device Laboratory, Des Plaines, IL, USA) and stored at −80 °C until further characterization.

### 2.4. Enterobacteriaceae and E. coli Confirmation

An indole spot test was used to distinguish among Enterobacteriaceae genera and species and confirm isolates as *E. coli*. Isolates were inoculated onto TSA (BD Difco™, Franklin Lakes, NJ, USA) and incubated for 18–24 h at 37 °C. A single colony was smeared on Whatman filter paper and saturated with 1–2 drops of James reagent (bioMérieux, Marcy l’Etoile, France). Twenty-six isolates negative for the indole test were confirmed as *E. coli* using Microflex^®^ LRF (Bruker Daltonics GmbH, Leipzig, Germany), a matrix-assisted laser desorption ionization-time of flight mass spectrometry (MALDI-TOF MS) using previously published methods [13]. Isolates were considered as ESBL-producing *E. coli* if MALDI-TOF or indole results were positive for *E. coli* and *bla*_CTX-M_ presence was confirmed by PCR and gel electrophoresis.

### 2.5. E. coli Enumeration by Colony Forming Units per Gram Feces (CFU/g)

Ten samples were conveniently chosen from each age group, based on the availability of glycerol stock after enrichment, to determine the CFU/g feces of *E. coli* and antibiotic-resistant *E. coli.* A total of 30 samples across all age groups were processed. Processing began by diluting 0.5 g of fecal glycerol stock into 4.5 mL of 1× Gibco^®^ phosphate buffered saline (PBS) (Thermo Fisher Scientific, Gaithersburg, MD, USA), this resulted in a 1:20 dilution factor. A serial 1:10 dilution of all samples was completed by transferring 0.5 mL of PBS into 4.5 mL fresh PBS, resulting in 1:200 and 1:2000 dilution factors. Next, 50μL of the PBS sample dilution factors 1:20, 1:200, or 1:2000 were spiral-plated using the Autoplate^®^ Spiral Plating System (Advanced Instruments, Norwood, MA, USA) onto MacConkey, MacConkey with 1 µg/mL ciprofloxacin, MacConkey with 32 µg/mL erythromycin, MacConkey with 16 µg/mL tetracycline, and MacConkey with 4 µg/mL ceftriaxone agars. Antibiotic concentrations for ceftriaxone, ciprofloxacin, and tetracycline were chosen based on CLSI (Clinical Laboratory Standards Institute) minimum inhibitory breakpoints for *E. coli* [26]. Erythromycin concentration was used at the same concertation as the CDC National Antimicrobial Resistance Monitoring System (NARMS) for enteric bacteria interpretive standard for azithromycin (32 µg/mL) since no CLSI minimum inhibitory breakpoint for *E. coli* has been determined [27]. Dilution factors plated for each media were chosen based on a test run of one sample from each age group, where countable colonies were present at a 1:200 dilution for MacConkey, MacConkey with 32 µg/mL erythromycin, and MacConkey with 16 µg/mL tetracycline; MacConkey with 4 µg/mL ceftriaxone and MacConkey with 1 µg/mL ciprofloxacin agars had countable colonies at 1:20. Plates were incubated for 18–24 h at 37 °C, and *E. coli* CFU/g feces were determined by counting pink, lactose fermenting colonies using the automated Flash and Go system (IUL, Barcelona, Spain). If too few colonies were present (< 10), samples were re-plated at a lower dilution factor and if too many colonies were present (> 300) samples were re-plated at a higher dilution factor. Raw CFU/g feces *E. coli* were log_10_ transformed for all age groups and media types. The difference between total *E. coli* and resistant *E. coli* was calculated from the difference in log_10_ CFU/g feces on MacConkey and on MacConkey supplemented with either tetracycline, erythromycin, ceftriaxone, or ciprofloxacin; of note, a higher difference corresponded to a lower antibiotic-resistant *E. coli* enumeration.

### 2.6. DNA Extraction and Endpoint PCR for bla_CTX-M_, qnrB, and mph(A) Genes

An isolated colony was selected from TSA with 5% Sheep Blood (BD Difco™, Franklin Lakes, NJ, USA) and dissolved in 50 µL Invitrogen™ UltraPure™ DNase/RNase-Free Distilled Water (Thermo Fisher Scientific, Waltham, MA, USA). DNA was extracted by boiling for 10 min at 95 °C. DNA quality was assessed at the 260/280 nm ratio of absorbance and quantity in ng/µL was determined using a Fluostar Omega microplate reader and LVis Plate (BMG LabTech Inc., Cary, NC, USA). DNA was stored at 4 °C during experiments and −30 °C once experiments were completed. Polymerase chain reaction (PCR) for isolates was performed using the Eppendorf Mastercycler^®^ nexus thermocycler (Eppendorf AG, Enfield, CT, USA) for each gene under their respective conditions. Reactions were carried out with GoTaq^®^ G2 Hot Start Master Mix and Nuclease-Free Water (Promega Corporation, Madison, WI, USA), following manufacturer recommendations for a 25 µL reaction with 2 µL of template DNA. PCR primer pairs were used to amplify genes conferring resistance to macrolides (*mph*(A)), plasmid-mediated fluoroquinolones (*qnrB*), and beta-lactamases (*bla*_CTX-M-Group-1_ and *bla*_CTX-M-Group-9_) (Integrated DNA Technologies, Inc., Coralville, IA) (Table 1). A lack of specificity with *qnrB* prompted an additional PCR reaction with *qnrB19*-specific primers to confirm resistance gene presence or absence; these primers were designed using PrimerQuest™ Tool (Integrated DNA Technologies, Inc., Coralville, IA, USA) using sequences from bacteria isolates encoding *qnrB19*. PCR conditions for all primer pairs underwent 30–35 cycles of denaturing at 94–95 °C for 1 min, annealing at primer specific temperature for 1 min, elongation at 72 °C for 1 min, and a final elongation step at 72 °C for 5 min. When available, previously published protocols were used for each primer pair (Table 1). Using the Lonza Flashgel™ System (Lonza Group Ltd., Basel, Switzerland), gel electrophoresis at 275 volts and 50 mA current for 3–5 min confirmed the presence or absence of resistance gene amplicons at the correct product size. Negative and positive controls, containing *mph*(A), *qnrB*, *bla*_CTX-M_ variant group 1, or *bla*_CTX-M_ variant group 9, were run in tandem with ESBL-producing *E. coli* isolates in both PCR reactions and gel electrophoresis.

### 2.7. Missing or Excluded Samples and Isolates

One sample from the yearling group showed phenotypic ESBL-producing growth on CHROM-ESBL agar and MacConkey agar with cefepime, but the presence of *bla*_CTX-M_ was not confirmed by PCR and gel electrophoresis; therefore, this sample and isolates were excluded from analysis. Additionally, two hutch calves were excluded from analysis due an age greater than 90 days, classifying these calves as outliers when compared with other calves within the hutch age group. Last, one sample in the weaned group was missing from the saved glycerol stocks and not enriched for ESBL-producing *E. coli*.

### 2.8. DNA Extraction and E. coli Whole-Genome Sequencing (WGS)

Eight isolates were chosen for further analysis by whole-genome sequencing: specifically, four isolates with each resistance genotype to *bla*_CTX-M_, *qnrB*, and *mph*(A) identified by PCR and gel electrophoresis from each of the two *bla*_CTX-M_ groups (*bla*_CTX-M_ variant group 1 and *bla*_CTX-M_ variant group 9) across the three age groups. Genomic DNA was extracted from the eight isolates on the fully automated QIAcube system using a QIAamp DNA Mini Kit (Qiagen, Valencia, CA, USA). Isolate DNA quality was assessed at the 260/280 nm ratio of absorbance, and quantity in ng/µL was determined using a Fluostar Omega microplate reader and LVis Plate (BMG LabTech Inc., Cary, NC, USA). Additionally, DNA was quantified following manufacturer instructions for the Qubit dsDNA HS (High Sensitivity) assay kit in a Qubit 4 fluorometer (Life Technologies, Carlsbad, CA, USA). Sequencing libraries were prepared according to manufacturer instructions using the Swift 2S™ Turbo DNA Library Kit and Swift Normalase™ Kit (Swift Biosciences, Ann Arbor, MI, USA). The quantity (ng/µL) of indexed libraries was measured using the Qubit dsDNA HS assay kit in a Qubit 4 fluorometer (Life Technologies, Carlsbad, CA, USA) and indexed library fragment size was measured using the Standard Sensitivity NGS fragment analysis kit and Fragment Analyzer automated CE system (Advanced Analytical, Des Moines, IA, USA). Indexed library quantity and fragment size were used as quality controls, ensuring that the concentration of DNA was not below 12 ng/µL and fragment sizes were within an acceptable range (500–600 bp), before library pooling. Sequencing was carried out on an Illumina MiSeq platform using a MiSeq Reagent Kit v2 (500-cycles) with 250 paired-end reads (Illumina, Inc San Diego, CA, USA) according to manufacturer instructions.

### 2.9. Bioinformatics Analysis

Command-line tools on the web-based Texas A&M University High-Performance Research Computer (HPRC) were used for the analysis of raw sequencing reads. Sequencing adapters were removed from raw-reads using Trimmomatic v.0.39 [31]. FastQC v.0.11.9 determined the quality of raw-reads, and reports were combined with MultiQC v.1.7 [32]. SPAdes v.3.14.0 was used to assemble genomes, and the quality of assemblies was assessed using the Quality Assessment Tool for Genome Assemblies (QUAST v.5.0.2) [33,34]. SerotypeFinder 2.0 was used to determine O:H serotypes of *E. coli* from FASTA files [35]. Sequence types (ST) were determined using an MLST allelic database of seven housekeeping genes (*adk*, *fumC*, *gyrB*, *icd*, *mdh*, *purA*, and *recA*) from raw sequencing reads using Bio-MLST-Check v.2.1.17 [36]. Resistance genes were identified using ABRicate v. 0.9.9 ResFinder from raw reads (minimum coverage = 60, threshold identity = 90), virulence genes were identified using virulence factor database (VFDB) (minimum coverage = 60, threshold identity = 90), and plasmids were identified using PlasmidFinder (minimum coverage = 80, threshold identity = 90) [37,38,39]. Chromosomal point mutations were identified using PointFinder, all found on the web-based Center for Genomic Epidemiology (https://cge.cbs.dtu.dk/services/ accessed 12 June 2021) [40].

### 2.10. Data Analysis

All statistical analyses were conducted using Stata statistical software: Release 17 [41]. Bivariable logistic regression was used to estimate associations between ESBL-producing *E. coli* prevalence, age, antibiotic treatments including prophylactic treatment (IPU), antibiotic treatments excluding prophylactic treatment (EPU), and health events requiring treatment (e.g., respiratory or bloat events). Antibiotic use was classified as binary (treated or untreated), regardless of the number of treatments due to low numbers of calves having two or more treatments in each antibiotic class. Log_10_ transformation of CFU/g feces growth was performed for normalization purposes and comparison of results; thereafter, enumerated *E. coli* on antibiotic selective MacConkey agars were subtracted from enumerated *E. coli* on non-selective MacConkey agar to calculate the antibiotic resistance difference in log_10_ CFU/g feces where smaller difference corresponds to increased antibiotic-resistant growth. A bivariable linear regression analysis was used to investigate calf age, antibiotic use, health events requiring treatment, and ESBL-producing *E. coli* presence with *E. coli* growth for log_10_ CFU/g feces and the antibiotic resistance difference in log_10_ CFU/g feces. A one-way ANOVA was performed on log_10_ CFU/g feces *E. coli* and the antibiotic resistance difference in log_10_ CFU/g feces *E. coli* to evaluate age and media types for total and resistant *E. coli* growth. Additionally, bivariable logistical regression was run for ESBL-producing *E. coli mph*(A) and *qnrB* prevalence for *bla*_CTX-M_ group. For all analyses, a *p*-value of 0.05 and below (*p* ≤ 0.05) was considered statistically significant. Graphics were coded in RStudio using R and R packages for data input and graphical generation [42,43,44,45,46,47,48,49].

### 2.11. Ethics Statement

Samples were collected under an animal use protocol (AUP) in accordance with the Animal Welfare Act, the Health Research Extension Act of 1985, and WTAMU Standard Operating Procedure 15.99.05.W1.02AR Institutional Animal Care and Use. The AUP (proposal # 2020.02.002) was approved by West Texas A&M University/Cooperative Research, Educational and Extension Team Institutional Animal Care and Use Committee (IACUC) on 10 February 2020.

## 3. Results

### 3.1. ESBL Prevalence and Antibiotic Use in Hutch, Weaned, and Yearling Calves

**Figure 1:**
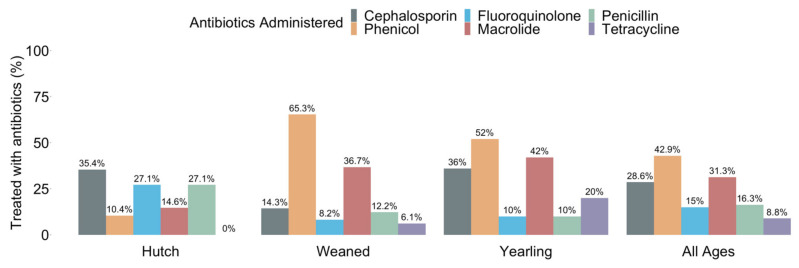
The percentage of calves treated at least once, due to health events requiring treatment, with cephalosporin, phenicol, fluoroquinolone, macrolide, penicillin, or tetracycline classes of antibiotics within each age group. Percentages represent the number of calves treated at least once within each antibiotic class over the total number calves in each age group. All 147 calves had antibiotic use data available from hutch (*n* = 48), weaned (*n* = 49), and yearling (*n* = 50) age groups. All calves received prophylactic penicillin and aminoglycoside treatments; weaned and yearling calves received prophylactic tetracycline treatment. Point estimates with 95% confidence intervals are provided in Table S2.

Calf age groups were categorized by housing location: hutch calves housed in individual hutches ranged from 1.6 to 3.0 months, weaned calves in group housing ranged from 3.5 to 6.9 months, and yearling calves in group housing ranged between 12 to 16.5 months of age (Table 2). Antibiotic administration data were available for all calves: there were 120 calves administered antibiotics due to health events, and the remaining 27 received only prophylactic antibiotics. In total, the most frequently administered antibiotic class across all age groups for health events requiring treatment was a phenicol (42.9%), followed by macrolides (31.3%), cephalosporins (28.6%), penicillins (16.3%), fluoroquinolones (16.0%), and tetracyclines (8.8%) (Figure 1). Of the 147 calves feces screened, across all age groups, 48.3% (95%CI 40.0–56.7%, *n* = 71) yielded positive ESBL-producing phenotypic results on CHROM-ESBL agar and MacConkey agar with cefepime, with isolates from these 71 samples confirmed by PCR to harbor the variant group 1 or 9 of the beta-lactamase gene *bla*_CTX-M_. Within the three age groups, prevalence of confirmed fecal ESBL-producing *E. coli* in the hutch group was 77.1% (95%CI 62.7–88.0%, *n* = 37), in the weaned group it was 61.2% (95%CI 46.2–74.8%, *n* = 30), and in the yearling group it was 8.0% (95%CI 2.2–19.2%, *n* = 4) (Table 2). Bivariable logistic regression analysis across all age groups showed no significant association between antibiotic treatment or health event requiring treatment and ESBL-producing *E. coli* prevalence for all variables except tetracycline treatment IPU (OR 0.16, *p* < 0.001) or EPU (OR 0.08, *p* = 0.015), and eye health events (OR 0.12, *p* = 0.050) (Table S1). Notably, all eye events received tetracycline as treatment, and only occurred in the weaned and yearling age groups. The significance of tetracycline treatment EPU was not different from tetracycline IPU. Additionally, calf age group was a significant predictor of ESBL-producing *E. coli* prevalence (OR 5.54, *p* < 0.001). The comparison among age groups showed a significantly higher prevalence of ESBL-producing *E. coli* in hutch and weaned calves when compared with the yearling calves, but no difference between hutch and weaned calves (Table S1).

### 3.2. Enumeration of Antibiotic-Resistant E. coli in Hutch, Weaned, and Yearling Calves

**Figure 2:**
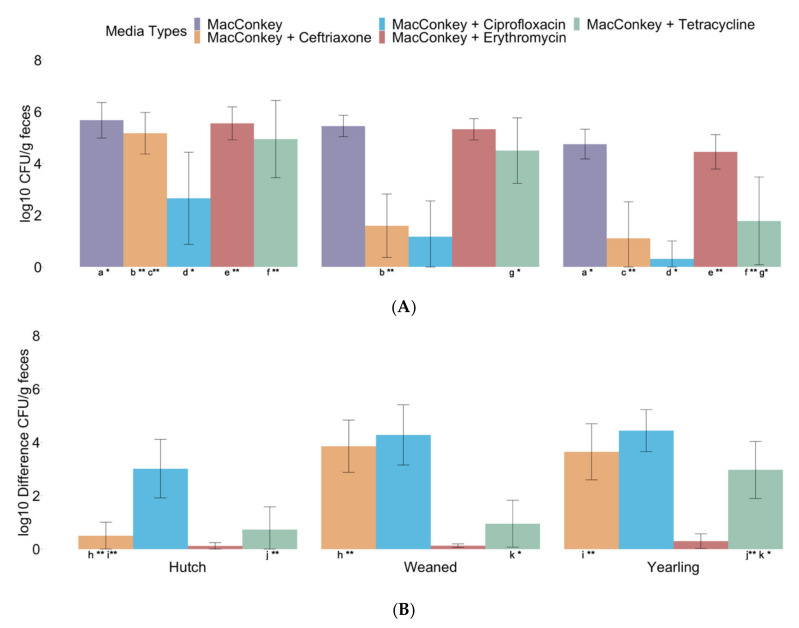
(**A**) log_10_ transformed CFU/g feces *E. coli* enumeration from hutch (*n* = 10), weaned (*n* = 10), and yearling (*n* = 10) age groups by media type (MacConkey, MacConkey with 4 µg/mL ceftriaxone, MacConkey with 1 µg/mL ciprofloxacin, MacConkey with 32 µg/mL erythromycin, and MacConkey with 16 µg/mL tetracycline agars). Significant differences between age groups are indicated by corresponding superscript letters and *p*-value (** *p* < 0.01 and * *p* < 0.05); ^a^ MacConkey (*p* = 0.048), ^b^ MacConkey with 4 µg/mL ceftriaxone (*p* < 0.001), ^c^ MacConkey with 4 µg/mL ceftriaxone (*p* < 0.001), ^d^ MacConkey with 1 µg/mL ciprofloxacin (*p* = 0.031), ^e^ MacConkey with 32 µg/mL erythromycin (*p* = 0.033), ^f^ MacConkey with 16 µg/mL tetracycline (*p* = 0.007), ^g^ MacConkey with 16 µg/mL tetracycline (*p* = 0.022). (**B**) Difference between MacConkey log_10_ transformed CFU/g feces and antibiotic medias (MacConkey with 16 µg/mL tetracycline, MacConkey with 32 µg/mL erythromycin, MacConkey with 4 µg/mL ceftriaxone, and MacConkey with 1 µg/mL ciprofloxacin agars) from hutch (*n* = 10), weaned (*n* = 10), and yearling (*n* = 10) age groups. Significant differences between age groups for the difference in CFU/g feces are indicated by corresponding superscript letters and *p*-value (** *p* < 0.01 and * *p* < 0.05); ^h^ MacConkey with 4 µg/mL ceftriaxone (*p* < 0.001), ^i^ MacConkey with 4 µg/mL ceftriaxone (*p* < 0.001), ^j^ MacConkey with 16 µg/mL tetracycline (*p* = 0.007), and ^k^ MacConkey with 16 µg/mL tetracycline (*p* = 0.017).

Younger calves in the hutch age group exhibited increased levels of *E. coli* log_10_ CFU/g in their feces compared with older calves in the weaned and yearling age groups. Hutch calves showed significantly higher levels of *E. coli* log_10_ CFU/g in their feces than weaned calves on media supplemented with ceftriaxone, as well as yearling calves on all media types: MacConkey, MacConkey with 16 µg/mL tetracycline, MacConkey with 32 µg/mL erythromycin, MacConkey with 4 µg/mL ceftriaxone, and MacConkey with 1 µg/mL ciprofloxacin (Figure 2A). Weaned calves showed significantly higher *E. coli* log_10_ CFU/g feces than yearling calves on MacConkey with 16 µg/mL tetracycline (Figure 2A). The difference between total and tetracycline-resistant *E. coli* log_10_ CFU/g was significantly higher in yearling calves, corresponding to less tetracycline-resistant *E. coli*, when compared with hutch and weaned calves (Figure 2B). Additionally, the difference between total and ceftriaxone-resistant *E. coli* log_10_ CFU/g was significantly lower in hutch calves, corresponding to more ceftriaxone-resistant *E. coli*, when compared with weaned and yearling calves (Figure 2B).

Bivariable linear regression analysis of *E. coli* enumeration across all ages for antibiotic treatments and health events requiring treatment showed significant relationships for ceftriaxone- and ciprofloxacin-selective agars (Table S3). Antibiotic treatment with phenicol (F(1, 28) = 5.51 Coef. = −1.94, *p* = 0.026) and tetracycline IPU (F(1, 28) = 36.72 Coef. = −3.82, *p* = < 0.001), as well as respiratory health events (F(1, 28) = 7.02, Coef. = −3.15, *p* = 0.013) were identified as significant predictors for cephalosporin-resistant *E. coli* log_10_ CFU/g feces on MacConkey supplemented with 4 µg/mL ceftriaxone. Antibiotic treatment with tetracycline IPU (F(1, 28) = 6.72, Coef. = −1.91 *p* = 0.015) was identified as a significant predictor of fluoroquinolone-resistant *E. coli* log_10_ CFU/g feces on MacConkey with 1 µg/mL ciprofloxacin. Antibiotic treatment with macrolides (F(1, 28) = 5.09, Coef. = −1.91 *p* = 0.032) was identified as a significant predictor of tetracycline-resistant *E. coli* log_10_ CFU/g feces on MacConkey with 16 µg/mL tetracycline. All other media types (MacConkey and MacConkey with 32 µg/mL erythromycin) showed no significant associations with any antibiotic treatments or health events requiring treatment. Additionally, bivariable linear regression analysis of ESBL-producing *E. coli* presence showed significant associations with *E. coli* CFU/g feces on MacConkey (F(1, 28) = 4.19, Coef. = 0.62, *p* = 0.050), MacConkey with 16 µg/mL tetracycline (F(1, 28) = 12.64, Coef. = 2.73, *p* = 0.001), and MacConkey with 32 µg/mL erythromycin (F(1, 28) = 6.32, Coef. = 0.85, *p* = 0.018) (Table S3).

The difference in total and antibiotic-resistant *E. coli* enumeration across all ages for antibiotic treatments IPU, antibiotic treatments EPU, health events requiring treatment, and ESBL-producing *E. coli* presence showed similar significant relationships as the log_10_ CFU/g feces enumeration of *E. coli* (Table S4). Treatment with phenicol (F(1, 28) = 8.93, Coef. = 2.00, *p* = 0.006), penicillin EPU (F(1, 28) = 5.06, Coef. −2.13, *p* = 0.033), and tetracycline IPU (F(1, 28) = 36.82, Coef. = 3.25, *p* < 0.001), as well as respiratory health events (F(1, 28) = 9.03, Coef. = 2.95, *p* = 0.006) and bloat health events (F(1, 28) = 5.06, Coef. = −2.13, *p* = 0.033) showed significant associations with the difference in *E. coli* CFU/g feces on MacConkey with 4 µg/mL ceftriaxone. Antibiotic treatment with cephalosporin (F(1, 28) = 6.06, Coef. = 0.25, *p* = 0.020) was significantly associated with a difference in *E. coli* CFU/g feces on MacConkey with 32 µg/mL erythromycin. Additionally, treatment with tetracycline IPU (F(1, 28) = 4.72, Coef. = 1.34 *p* = 0.038) was identified as a significant predictor of the difference in fluoroquinolone-resistant *E. coli* log_10_ CFU/g feces on MacConkey with 1 µg/mL ciprofloxacin and treatment with macrolide (F(1, 28) = 4.61, Coef. = 1.13, *p* = 0.040) was associated with the difference in tetracycline-resistant *E. coli* log_10_ CFU/g feces on MacConkey with 16 µg/mL tetracycline. ESBL-producing *E. coli* presence also showed significant associations with MacConkey with 16 µg/mL tetracycline (F(1, 28) = 15.70, Coef. = −2.11, *p* < 0.001) and MacConkey with 32 µg/mL erythromycin (F(1, 28) = 5.13, Coef. = −0.22, *p* = 0.031).

### 3.3. Resistance Profiles Identified by PCR for blaCTX-M, qnrB, and mph(A)

**Figure 3:**
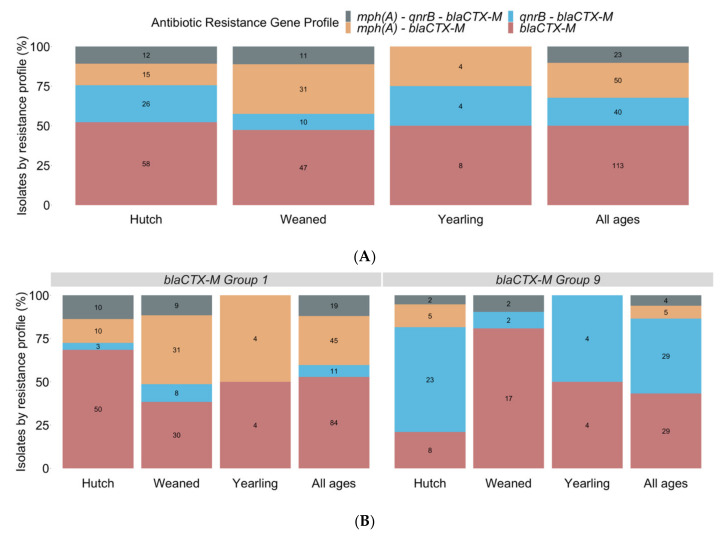
(**A**) Proportions of the four resistance gene profiles (*bla*_CTX-M,_ *mph*(A)*-bla*_CTX-M,_ *qnrB-bla*_CTX-M,_ or *mph*(A)*-qnrB-bla*_CTX-M_) identified by PCR and gel electrophoresis in all ESBL-producing *E. coli* isolates across all three age groups (hutch *n* = 111, weaned *n* = 99, yearling *n* = 16, and all ages *n* = 226). (**B**) Proportion of ESBL-producing *E. coli* isolates belonging to *bla*_CTX-M_ variant group 1 (*n* = 159) and *bla*_CTX-M_ variant group 9 (*n* = 67) across all three age groups (hutch, weaned, and yearling) by the four resistance gene profiles *bla*_CTX-M,_ *mph*(A)*-bla*_CTX-M,_ *qnrB-bla*_CTX-M,_ or *mph*(A)*-qnrB-bla*_CTX-M_.

In total, 226 ESBL-producing *E. coli* isolates were collected from 71 phenotypic ESBL-positive samples across all age groups. The isolates were confirmed as ESBL-producing by the presence of *bla*_CTX-M_ resistance gene by PCR and gel electrophoresis; however, other resistance genes (*bla*_SHV_ or *bla*_TEM_) may confer the ESBL-producing phenotype but were not screened for in this study [50]. An additional 4 *E. coli* isolates, collected from a sample in the yearling age group, harbored only the fluoroquinolone resistance gene *qnrB* but initially grew on CHROM-ESBL agar and MacConkey agar with cefepime, indicating ESBL activity. It is possible that a plasmid harboring the *bla*_CTX-M_ gene in these isolates was lost due to extended time on non-selective media; therefore, these isolates were excluded from analysis. Additional resistance genes to macrolides (*mph*(A)) and plasmid-mediated quinolones (*qnrB*) were also characterized by PCR and gel electrophoresis in all isolates (Figure 3A). In 113 isolates (hutch *n* = 58, weaned *n* = 47, yearling *n* = 8), the only antibiotic resistance gene present was *bla*_CTX-M_. In the remaining 113 isolates, in addition to *bla*_CTX-M_*, mph*(A) alone was present in 50 isolates (hutch *n* = 15, weaned *n* = 31, yearling *n* = 4), *qnrB* alone was present in 40 isolates (hutch *n* = 26, weaned *n* = 10, yearling *n* = 4), and both *mph*(A) and *qnrB* were present in 23 isolates (hutch *n* = 12, weaned *n* = 11, yearling *n* = 0) (Figure 3A).

Of the 226 isolates with *bla*_CTX-M_, 159 (70.4%) isolates belonged to *bla*_CTX-M_ variant group 1, and 67 (29.6%) isolates belonged to *bla*_CTX-M_ variant group 9 (Figure 3B). From the isolates in variant group 1, *mph*(A) was present in 45 isolates (hutch *n* = 10, weaned *n* = 31, yearling *n* = 4), *qnrB* was present in 11 isolates (hutch *n* = 3, weaned *n* = 8, yearling *n* = 0), and both *mph*(A) and *qnrB* were present in 19 isolates (hutch *n* = 10, weaned *n* = 9, yearling *n* = 0). From the isolates in variant group 9, *mph*(A) was present in 5 isolates (hutch *n* = 5, weaned *n* = 0, yearling *n* = 0) *qnrB* was present in 29 isolates (hutch *n* = 23, weaned *n* = 2, yearling *n* = 4), and both *mph*(A) and *qnrB* were present in 4 isolates (hutch *n* = 2, weaned *n* = 2, yearling *n* = 0) (Figure 3B). Based on bivariable logistic regression analysis, across all age groups, isolates with *mph*(A) (OR 4.34, *p* < 0.001) were more commonly associated with *bla*_CTX-M_ variant group 1 than isolates with *qnrB* (OR 0.24, *p* < 0.001). Inversely, isolates with *qnrB* (OR 4.17, *p* < 0.001) had a higher association with *bla*_CTX-M_ variant group 9 than isolates with *mph*(A) (OR 0.23, *p* < 0.001) (Table S5).

### 3.4. WGS of ESBL-Producing E. coli Isolates

From the eight sequenced isolates, *bla*_CTX-M_ variants *bla*_CTX-M-1_, *bla*_CTX-M-15_, and *bla*_CTX-M-32_ were present in one isolate each, *bla*_CTX-M-27_ and *bla*_CTX-M-65_ were present in two isolates each, and *bla*_CTX-M-102_ and *bla*_CTX-M-174_ were both identified with low sequence coverage in the same single isolate (Table 3). The diversity of isolates was not limited to the variation of *bla*_CTX-M_, O:H serotypes (O4:H11, O8:H30, O26:H11, O40:H4, O70:H2, O100:H30, O103:H2, and O134:H38) and sequence types were different for each isolate (ST10, ST29, ST58, ST154, ST226, ST641, ST993, and ST1967). Antibiotic resistance genes (ARGs) previously identified by PCR and gel electrophoresis were confirmed in all isolates, with additional macrolide, fluoroquinolone, and beta-lactam resistance genes observed. All eight isolates encoded the MLS (macrolide, lincosamide, and streptogramin) resistance gene *mdf*(A), two isolates encoded PMQR genes *qnrA1* and *qnrS1*, and three isolates also harbored the beta-lactamase genes *bla*_TEM-1_ and *bla*_CARB-2_. Other ARG classes were also identified in all isolates; four isolates had tetracycline resistance genes (*tet*(A)), four isolates had phenicol resistance genes (*floR*), seven isolates had sulfonamide resistance genes (*sul1* or *sul2*), and five isolates had aminoglycoside resistance genes (*aph(3′)-IIa, aph(6)-Id, aph(3″)-Ib or aph(3′)-Ia*). Additionally, plasmids from replicon incompatibility (Inc) groups R, Y, FIB, FIA, FII, HI2, and I1α were identified across all eight sequenced isolates, as well as three Col plasmids (Table 3).

## 4. Discussion

### 4.1. Age-Related Prevalence of ESBL-Producing E. coli

The prevalence of ESBL-producing *E. coli* in the hutch, weaned, and yearling-age groups varied considerably. Overall, age was a significant predictor for ESBL-producing *E. coli* presence and upon further analysis, significant differences between the oldest calves in the yearling group and the younger calves in the hutch and weaned groups were identified (Table S2). The observed elevation of ESBL-producing *E. coli* prevalence in younger calves is consistent with other studies showing an inverse relationship between prevalence levels of antibiotic-resistant *E. coli* and increasing age [8,25]. These results suggest that hutch and weaned dairy calves may be potent reservoirs for ESBL-producing *E. coli.* Importantly, this decreases with age, reducing the risk of animals as they approach joining the milking herd or slaughter age. The previous use of phenicol, macrolide, cephalosporin, fluoroquinolone, penicillin or tetracyclines antibiotic classes for either treatment or prophylaxis did not positively impact the observed fecal prevalence of ESBL-producing *E. coli.* The only relationship observed between antibiotic use and ESBL-producing *E. coli* prevalence was protective; calves treated with tetracycline IPU were less likely to harbor ESBL-producing *E. coli* than untreated calves. Results indicate that prophylactic use did not impact the protective relationship between tetracycline administration and ESBL-producing *E. coli* in the weaned and yearling age groups on this calf-raising farm. Potential associations between antibiotic use and ESBL-producing *E. coli* prevalence were limited due to the small total sample size, within age group sample size, and the single collection of samples. Additional studies to detect the cumulative effects of antibiotic treatments within and among animals, and antibiotic resistance in the young dairy calves and their environment, are needed to identify the direct risk of environmental cross-contamination and monitor changes as calves age, are treated with antibiotics, or else change housing environments.

### 4.2. Antibiotic Use and Age-Related Growth of E. coli and Antibiotic-Resistant E. coli

Hutch calves showed higher levels of *E. coli* log_10_ CFU/g in their feces than the weaned and yearling calves, indicating an inverse relationship between age and *E. coli* growth in calf feces (Figure 2A). Significant differences in log_10_ CFU/g enumeration on MacConkey and MacConkey agar supplemented with antibiotics (i.e., erythromycin, tetracycline, ciprofloxacin, or ceftriaxone) suggest that the quantity of *E. coli* and *E. coli* resistant to these antibiotics is greatest in the youngest hutch calves. Younger calves typically are at increased risk of disease, often requiring antibiotic treatment, additionally these calves received multiple prophylactic antibiotics on this farm as a part of calf management practices. Growth on MacConkey agar and MacConkey agar with all antibiotic types was significantly higher for hutch calves compared with yearling calves. Additionally, hutch calves also displayed a higher quantity of ceftriaxone-resistant *E. coli* than weaned calves. Weaned calves displayed a higher quantity of tetracycline-resistant *E. coli* than yearling calves; however, weaned calves would have received tetracycline more recently than yearling calves; therefore, this relationship may be impacted by calf management and prophylactic treatment protocols. Similar to log_10_ CFU/g feces, the difference in total *E. coli* growth from antibiotic-resistant *E. coli* growth showed less difference in between non-selective and antibiotic selective CFU/g feces for the hutch calves (Figure 2B). Increased ceftriaxone-resistant *E. coli* CFU/g feces, corresponding to a lower difference in CFU/g feces, was observed for hutch calves when compared with weaned and yearling calves. A higher difference in CFU/g feces, showed tetracycline-resistant *E. coli* CFU/g feces was significantly lower in yearling calves compared with hutch and weaned calves. Again, the difference between tetracycline-resistant *E. coli* growth in yearling and weaned calves may be influenced by the more recent use of prophylactic tetracycline in weaned calves.

Bivariable linear regression analysis for all age groups identified significant associations between ESBL-producing *E. coli* presence and increased *E. coli* concentration on media supplemented with erythromycin and tetracycline, as well as non-antibiotic selective media for the log_10_ CFU/g only. These results indicate that samples with ESBL-producing *E. coli* may have overall higher numbers of both antibiotic-resistant and non-resistant bacteria. Additionally, respiratory health events were associated with decreased ceftriaxone-resistant growth, while bloat health events were associated with an increased difference in ceftriaxone-resistant growth. Health events can correspond to antibiotic treatment protocols, or specific use of antibiotic classes (e.g., tetracycline use in all eye health events); therefore, these factors are important to consider when planning for research regarding antibiotic treatment. Across all age groups, analysis revealed treatment with phenicol for a health event or tetracycline IPU were significant predictors of decreased *E. coli* growth on media supplemented with ceftriaxone. These results suggest that the quantity of *E. coli*, as well as antibiotic-resistant *E. coli*, is lowest in yearling calves and highest in hutch calves. However, the small sample size limits the results of this study. A larger analysis is required to better explore associations between antibiotic-resistant *E. coli* quantity, age, and antibiotic treatment. Regardless, these observations are compelling, and are corroborated by other studies showing an increased level of antibiotic-resistant *E. coli* in young dairy calves compared with older age groups calves on this farm with management and treatment practices in place.

Antibiotics used to enumerate antibiotic-resistant *E. coli* (erythromycin, tetracycline, ciprofloxacin, or ceftriaxone) were chosen for their clinical relevance in human or veterinary medicine. Ceftriaxone, a third-generation cephalosporin similar to ceftiofur used in cattle, alone does not exclusively screen for ESBLs; that is, it is an indicator of ESBL and Amp*C* beta-lactam resistance and above (e.g., carbapenemases) [51,52]. Therefore, ESBL-producing *E. coli* cannot be enumerated, but may be assumed as part of the *E. coli* population based on presence in all three calf age groups. Use of tetracycline in the form of oxytetracycline is common in veterinary medicine either for treatment of disease or as a prophylactic, resulting in a wide array of tetracycline resistance genes, often efflux pumps (e.g., *tet*(A)), observed in *E. coli* as well as other enteric pathogens [53]. Management protocols for the dairy calves in this study included both treatment for health events or prophylactic use of oxytetracycline, potentially impacting any relationships observed for differences in tetracycline resistance between age groups or tetracycline use in relation to *E. coli* growth. Ciprofloxacin is an active metabolite of enrofloxacin, both of which are fluoroquinolone antibiotics [54]. Enrofloxacin used in veterinary medicine, specifically in dairy calves for treatment of respiratory disease [55]. As expected, minimal growth was observed on agar supplemented with ciprofloxacin at 1 µg/mL in all age groups at the current CLSI breakpoint, since PMQR genes do not confer total resistance to quinolones but tend to confer reduced susceptibility (e.g., at 0.25 or 0.5 µg/mL). Tetracycline, ciprofloxacin, and ceftriaxone all have minimum inhibitory breakpoints for *E. coli*, but erythromycin does not [26]. A lack of CLSI breakpoints established for macrolide antibiotics (e.g., azithromycin or erythromycin) in *E. coli* can be attributed to the inherent resistance of *E. coli* to erythromycin through multiple mechanisms [16,56,57]. Tylosin, tulathromycin, and tildipirosin were the commonly administered macrolide antibiotics for treatment of respiratory health events in these dairy calves [22,58,59]. Erythromycin was chosen for *E. coli* enumeration because the macrolide resistance gene of interest in this study, *mph*(A), encodes for the resistance enzyme MPH(2′)-I, which preferentially inactivates 14-membered macrolides (e.g., erythromycin) over 15-membered macrolides (e.g., azithromycin or tulathromycin) and 16-membered macrolides (e.g., tylosin) [60]. Additionally, other studies have shown that high concentrations erythromycin may inhibit *E. coli* growth [56]. Agar supplemented with 32 µg/mL erythromycin showed consistently high growth of *E. coli* across all three age groups. Erythromycin-resistant growth was comparable to *E. coli* growth on non-selective agar, indicative of high levels of *E. coli* resistant to erythromycin in this population of dairy calves. While erythromycin-resistant *E. coli* may harbor other macrolide resistance genes, *mph*(A) is most likely present in a portion of the erythromycin-resistant *E. coli* population, similar to the observed *mph*(A) prevalence in ESBL-producing *E. coli* across all three calf age groups.

### 4.3. Genotypic Resistance Profiles Identified by PCR for bla_CTX-M_, qnrB, and mph(A)

Macrolide, fluoroquinolone, and cephalosporin resistance gene profiles were identified via PCR and gel electrophoresis of antibiotic resistance genes *mph*(A)*, qnrB,* and two variant groups of *bla*_CTX-M_. Four resistance gene profiles (*bla*_CTX-M_, *mph*(A)*-bla*_CTX-M_, *qnrB-bla*_CTX-M_, or *mph*(A)*-qnrB-bla*_CTX-M_) were identified for the 226 ESBL-producing *E. coli* isolates. Whole-genome sequencing confirmed the presence of *mph*(A)*, qnrB,* and *bla*_CTX-M_ genes in eight isolates. However, one isolate classified as the *bla*_CTX-M_ variant group 1 via PCR and gel electrophoresis was identified as variant group 9 variant *bla*_CTX-M-27_, of which the original sample contained isolates positive for *bla*_CTX-M_ variant group 1 and variant group 9 suggesting a heterogeneous mix of ESBL-producing *E. coli* subtypes within at least one sample. Across all age groups, the most common resistance gene profile identified was *bla*_CTX-M_ alone. The lack of additional macrolides and fluoroquinolones resistance genes in many of the ESBL-producing *E. coli* in these calves indicates weak selective pressures, if any, are present for co-resistance of cephalosporin and macrolide or fluoroquinolone genes on this calf-raising farm at the time samples were taken. The potential remains for other genes conferring additional resistance patterns (e.g., *floR* or *tet*(A)) to a variety of antibiotic classes, as seen in the whole-genome sequenced isolates. However, the limited array of PCR assays conducted in this study did not screen for additional macrolide, fluoroquinolone, or other resistance genes limiting this study. In the remaining isolates, ESBL-producing *E. coli* with macrolide or fluoroquinolone resistance genes were observed by resistance gene profiles *mph*(A)*-bla*_CTX-M_, *qnrB-bla*_CTX-M_, or *mph*(A)*-qnrB-bla*_CTX-M_ (Figure 3A). In hutch calves_,_ more isolates with the resistance gene profile *qnrB-bla*_CTX-M_ were present than isolates with *mph*(A)*-bla*_CTX-M_. However, in the weaned calves more isolates with the resistance gene profile *mph*(A)*-bla*_CTX-M_ were present than isolates with *qnrB-bla*_CTX-M_. Notably, a similar number of isolates with the resistance gene profile *mph*(A)*-qnrB-bla*_CTX-M_ were identified in both the hutch and weaned calves, showing no age discrimination between groups for presence of macrolide, fluoroquinolone, and cephalosporin resistance genes. Yearling calves showed equal amounts of the resistance gene profiles of *mph*(A)*-bla*_CTX-M_ and *qnrB-bla*_CTX-M_ and had no isolates with the gene combination *mph*(A)*-qnrB-bla*_CTX-M_. The composition of resistance gene profiles varied between age groups, with *mph*(A) presence highest in ESBL-producing *E. coli* isolates from weaned calves and *qnrB* presence highest in ESBL-producing *E. coli* isolates from hutch calves. This observation is consistent with antibiotic administration data for health events in hutch and weaned calves which shows a higher percentage of hutch calves were treated with fluoroquinolones while a higher percentage of weaned calves were treated with macrolides (Figure 1). Unfortunately, the measures of association between isolate resistance gene profile, calf age group, or antibiotic use were not conducted due to the study design’s criteria limiting the collection of isolates from ESBL-producing positive samples, and the *mph*(A) or *qnrB* prevalence not measured among non-ESBL-producing *E. coli*.

While study design, single collection of samples, and the small sample size of the isolates with each resistance gene profile were additional limitations in determining relationships between age group and additional antibiotic resistance genes present in ESBL-producing *E. coli*, results show varying affinities for the co-resistance of macrolide, fluoroquinolone, and cephalosporin antibiotic resistance genes at differing calf ages on this farm. In addition to the identification of macrolide, fluoroquinolone, and cephalosporin resistance gene profiles, ESBL-producing *E. coli* isolates were further differentiated by *bla*_CTX-M_ variant group. Variants within *bla*_CTX-M_ belong to six specific groups, or sub-lineages; group 1 variants include but are not limited to CTX-M-1 and CTX-M-15, while group 9 variants include but are not limited to CTX-M-14 and CTX-M-27 [61,62]. All isolates were identified as either the *bla*_CTX-M_ variant group 1 or variant group 9 by PCR and gel electrophoresis (Figure 3B). These CTX-M groups contain some of the most common variants identified in dairy cattle (e.g., *bla*_CTX-M-15_, *bla*_CTX-M-32_, *bla*_CTX-M-27_, or *bla*_CTX-M-65_) [10,63]. Bivariable logistic regression analysis revealed a significant relationship between resistance gene profile and *bla*_CTX-M_ group (Table S5). The macrolide resistance gene *mph*(A) had a higher association with *bla*_CTX-M_ variant group 1, while the fluoroquinolone resistance gene *qnrB* was more likely to be associated with *bla*_CTX-M_ variant group 9, suggesting the potential preference for co-resistance gene profile based on the *bla*_CTX-M_ variant group. This may be explained through plasmid co-location or other mobile genetic elements located near gene pairs, neither of which were investigated or identified as a part of this study. Because calves may differ in origin farm, any difference in the association of *bla*_CTX-M_ variant groups with *qnrB* and *mph*(A) genes could be affected by geographic location. Notably, overall, more isolates were identified as *bla*_CTX-M_ variant group 1 than variant group 9, a trend similar to other observed *bla*_CTX-M_ variant patterns in dairy cattle [64]. These antibiotic resistance gene patterns by the *bla*_CTX-M_ variant group suggest that variant group is a factor to consider when understanding the co-resistance of macrolide, fluoroquinolone, and cephalosporin antibiotics, and should be further explored. Further characterization of *bla*_CTX-M_ variants by group with additional antibiotic resistance patterns, and the plasmids or mobile genetic elements associated with them, would provide a better understanding of co-resistance genes.

### 4.4. Diversity of ESBL-Producing E. coli

Sequencing data from the eight *E. coli* isolates revealed a diverse range of *bla*_CTX-M_ variants, serogroups, and sequence types similar to other studies that have characterized *E. coli* harboring *bla*_CTX-M_ in dairy cattle [63,64]. The additional macrolide, PMQR, and beta-lactamase genes identified (*mdf*(A), *qnrA1*, *qnrS1*, *bla*_TEM-1_, and *bla*_CARB-2_) suggest there is the potential for a diverse range of macrolide, fluoroquinolone, and cephalosporin antibiotic resistance gene patterns in these dairy calves at this calf-raising farm. Resistance genes to tetracycline, phenicol, sulfonamide, aminoglycoside, and trimethoprim antibiotic classes were also identified in the sequenced isolates. The variety of antibiotic resistance genes observed, within multiple drug classes, may add difficulty to future surveillance of macrolide, fluoroquinolone, and cephalosporin resistance by expanding the combinations of genes for co-resistance. All sequenced ESBL-producing *E. coli* isolates were multidrug-resistant (resistant to three or more antibiotic classes); however, no common resistance pattern was identified among the eight isolates, likely due to the small number of isolates sequenced. The multidrug resistance in these isolates is a potential artifact of selection for isolates with *bla*_CTX-M_ and should not be extrapolated to all *E. coli* within this population. Notably, two serogroups, O103 and O26, were identified as non-O157 enterohemorrhagic *E. coli* of potential public health interest, with the O103 isolate encoding the shiga toxin producing gene *stx1* [65]. Four of the sequence types identified (ST10, ST29, ST58, and ST154) have been previously identified as *bla*_CTX-M_ encoding ESBL-producing *E. coli* [63]. Sequence type 29 has previously been associated with pathogenic strains of *E. coli*; however, the strain in this study lacks specific molecular characteristics of pathogenicity (e.g., shiga toxin production) [66]. Additionally, a wide variety of plasmid families associated with ESBL-producing *E. coli* antimicrobial resistance and virulence were identified in the sequenced isolates [67]. The consistency among sequence types and plasmid incompatibility groups suggest identifiable molecular characteristic of ESBL-producing *E. coli* isolated from dairy cattle and calves. While these isolates represent one farm at a single point in time, the range of *bla*_CTX-M_ variants, serogroups, sequence types, and ARGs present shows the potential variety for multi-drug resistance and potentially pathogenic ESBL-producing *E. coli* in dairy calves. Unfortunately, due to the low number of isolates sequenced, no associations could be determined between age groups, *bla*_CTX-M_ variant, serogroups, or antibiotic resistance gene profiles. Importantly, the observed diversity in ESBL-producing *E. coli* could be due to the variety of dairy farms these calves originated from, later to be reared at a single calf-raising farm.

## 5. Conclusions

The prevalence of ESBL-producing *E. coli* in dairy calves at this farm varied significantly by age. Young calves in hutch and weaned age groups had a higher prevalence of ESBL-producing *E. coli* than older yearling calves, but antibiotic use within these same calves was not a predictor of the sample-level prevalence of ESBL-producing *E. coli*. However, a protective relationship observed for tetracycline use was significant both with and without accounting for prophylactic use. Calf age along with certain antibiotic use and health events requiring treatment were significant covariates affecting log_10_ CFU/g feces of *E. coli* and antibiotic-resistant *E. coli*, as well as the resistance difference in log_10_ CFU/g feces of antibiotic-resistant *E. coli* in calf feces. Resistance gene profiles for macrolide, fluoroquinolone, and cephalosporin antibiotics were identified by PCR for the *bla*_CTX-M_*, qnrB,* and *mph*(A) genes and revealed a significant relationship between the *bla*_CTX-M_ variant group (1 or 9) and *qnrB* or *mph*(A) presence. Sequenced isolates showed a diverse range of ESBL-producing *E. coli* differing by *bla*_CTX-M_ variant, serogroup, sequence type, and additional antibiotic resistance genes. This study confirmed a high sample-level prevalence of ESBL-producing *E. coli* in the feces of dairy calves, with co-resistance of fluoroquinolones or macrolides resistance genes, and identified potential selection factors for multidrug-resistant ESBL-producing *E. coli* in dairy calves. These findings will be of potential importance to human health, further solidifying the need to understand how age, antibiotic use, and molecular characteristics of isolates can impact genotypic co-resistance to macrolide, fluoroquinolone, and cephalosporin antibiotics in dairy calves. Calf-raising farms can have calves originating from many farms and varying management practices therefore these data may not be applicable to all dairy calf-raising farms. To fully understand the role of antibiotic use in the selection of ESBL and multidrug-resistant ESBL-producing *E. coli*, a larger study designed with longitudinal collections on the same group of calves accounting for pen movements, all documented treatments, or health events would provide a more thorough understanding of age-related ESBL-producing *E. coli* and the selection pressures required for emerging and changing co-resistance to macrolide, fluoroquinolone, and cephalosporin antibiotics in dairy calves.

## Figures and Tables

**Table 1:** PCR primers used in this study.

Target Gene	Primer Sequence	Product	Annealing	Source
*mph*(A)	F 5′-AAC TGT ACG CAC TTG C-3′ R 5′-GGT ACT CTT CGT TAC C-3′	837 bp	52 °C	[28]
*qnrB* (1–6)	F 5′ -GGM ATH GAA ATT CGC CAC TG-3′ R 5′ -TTY GCB GYY CGC CAG TCG AA-3′	264 bp	54 °C	[29]
*bla*_CTX-M-Group-1_	F 5′-GCG TGA TAC CAC TTC ACC TC-3′ R 5′-TGA AGT AAG TGA CCA GAA TC-3′	260 bp	55 °C	[30]
*bla*_CTX-M-Group-9_	F 5′-ATC AAG CCT GCC GAT CTG GTT A-3′ R 5′-GTA AGC TGA CGC AAC GTC TGC-3′	293 bp	55 °C	[31]
*qnrB*19	F 5′-CAC ATT GCG TGA CCA ATT-3′ R 5′-GAT GCC TGG TAG CTG TCT AAC-3′	90 bp	60 °C	This study

**Table 2:** Data collected from hutch, weaned, and yearling calf fecal samples.

Item	Hutch	Weaned	Yearling	Total
No. of Calves	48	49	50	147
Mean Age (Range) *	1.8 (1.6–3.0)	5.0 (3.5–6.9)	12.9 (12.0–16.5)	6.6 (1.6–16.5)
No. Confirmed ESBL	37	30	4	71
% ESBL (95%CI)	77.1% (62.7–88.0%)	61.2% (46.2–74.8%)	8.0% (2.2–19.2%)	48.3% (40.0–56.7%)
No. ESBL Isolates **	111	99	16	226

* In months. ** Isolates confirmed with indole spot test or MALDI-TOF as *E. coli* and ESBL-producing by PCR of Groups 1 and 9 of *bla*CTX-M.

**Table 3:** ESBL-producing *E. coli* whole-genome sequencing results for antimicrobial resistance genes, plasmid Inc groups, serotype, and sequence type.

Isolate	Age Group	Resistance Genes by Antibiotic Class									Serotype	Sequence Type (ST)	Plasmids	PCR Profile (*bla*_CTX-M_ Group)
		Beta-Lactam	Aminoglycoside	Trimethoprim	Sulfonamide	MLS	Phenicol	Quinolone	Tetracycline	Point Mutation				
18-H-6-*Ecoli*-Feb2020-2	weaned	*bla*_CTX-M-1_ *bla*_TEM-1A_	*aph(3″)-Ib* *aph(6)-Id*		*sul2*	*mdf*(A) *mph*(A)	*floR*	*qnrB19*	*tet*(A)		O40:H4	226	IncY, IncR, Col440I	*mph*(A), *qnrB, bla*_CTX-M_ (1)
19-H-6-*Ecoli*-Feb2020-1	weaned	*bla*_CTX-M-32_		*dfrA1*	*sul1*	*mdf*(A) *mph*(A)	*floR*		*tet*(A)		O134:H38	154	IncFIB, IncR	*mph*(A)*, bla*_CTX-M_ (1)
21-B-6-*Ecoli*-Feb2020-2	yearling	*bla*_CTX-M-102_ * bla_CTX-M-174_ *	*aph(6)-Id*		*sul2*	*mdf*(A)		*qnrB19*			O26:H11	29	IncFIA, IncFIB, Col(MG828), Col440I, Col156	*qnrB, bla*_CTX-M_ (9)
34-H-6-*Ecoli*-Feb2020-1	weaned	*bla*_CTX-M-15_ *bla*_TEM-1B_	*aph(6)-Id* *aph(3″)-Ib*		*sul2*	*mdf*(A)		*qnrB19* *qnrS1*			O4:H11	641	IncFIB, IncY, Col440I	*qnrB, bla*_CTX-M_ (1)
5-B-3-*Ecoli*-Feb2020-1	yearling	*bla*_CTX-M-27_	*aph(3″)-Ib* *aph(6)-Id*		*sul2*	*mdf*(A)	*floR*		*tet*(A)		O8:H30	58	IncFIA, IncFIB, IncFII	*bla*_CTX-M_ (9)
5-H-6-*Ecoli*-Feb2020-3	weaned	*bla*_CTX-M-27_				*mdf*(A)			*tet*(A)		O70:H2	10	IncFIB, IncFII	*bla*_CTX-M_ (1)
13-W-3-*Ecoli*-Feb2020-3	hutch	*bla*_CTX-M-65_ *bla*_CARB-2_ *bla*_TEM-1A_	*aph(3′)-IIa* *aph(6)-Id* *aph(3″)-Ib* *aph(3′)-Ia*		*sul1* *sul2*	*mdf*(A) *mph*(A)		*qnrA1*		*gyrA* p.S83L **	O100:H30	993	IncI1α, IncX1, IncHI2, IncHI2A	*mph*(A)*, bla*_CTX-M_ (9)
47-W-3-*Ecoli*-Feb2020-2	hutch	*bla*_CTX-M-65_		*dfrA1*	*sul1* *sul2*	*mdf*(A) *mph*(A) *	*floR*	*qnrB19*			O103:H2	1967	IncHI2A, IncHI2, IncFII, Col440I, Col156	*mph*(A)*, qnrB, bla*_CTX-M_ (9)

* Low coverage of PCR identified genes. ** Location of point mutation in *gyrA* conferring quinolone resistance. MLS (macrolide, lincosamide, and streptogramin).

## Data Availability

Sequencing data presented in this study are openly available from The National Center for Biotechnology Information (NCBI) (https://www.ncbi.nlm.nih.gov/). This data can be found under BioProject number PRJNA766656. All other data is contained within the article or supplementary material, available in Table S6.

## Supplementary Materials

The following are available online at https://www.mdpi.com/article/10.3390/microorganisms10020411/s1, Table S1. Odds ratios determined from bivariable logistic regression analysis of ESBL *E. coli* prevalence by age, antibiotic administration, and treatment events, Table S2. Percentages of calves treated with antibiotics for health events requiring treatment, all 147 calves had antibiotic use data available from hutch (*n* = 48), weaned (*n* = 49), and yearling (*n* = 50) age groups, Table S3. Bivariable linear regression for age, antibiotic treatments, and log10 CFU/g feces growth on MacConkey, MacConkey with 16µg/mL tetracycline, MacConkey with 32 µg/mL erythromycin, MacConkey with 4µg/mL ceftriaxone, and MacConkey with 1µg/mL ciprofloxacin agars, Table S4. Bivariable linear regression for age, antibiotic treatments, and difference of log10 CFU/g feces growth between MacConkey and MacConkey agars supplemented with antibiotics (erythromycin, tetracycline, ciprofloxacin, or ceftriaxone at same concentration as Table S3), Table S5. Odds ratios determined from bivariable logistic regression analysis of *mph*(A) and *qnrB* prevalence in ESBL *E. coli* isolates by *bla*_CTX-M_ variant group, Table S6. Whole genome sequencing data for ESBL *E. coli* isolates submitted to NCBI under BioProject number PRJNA766656.
