# Supplementary material for: Prevalence and Profiles of Antibiotic Resistance Genes mph(A) and qnrB in Extended-Spectrum Beta-Lactamase (ESBL)-Producing Escherichia coli Isolated from Dairy Calf Feces
Source: Microorganisms. 2022 Feb 10;10(2):411. doi: 10.3390/microorganisms10020411 (PMC8880659; doi:10.3390/microorganisms10020411)
Supplement: Supplementary file 1 [file microorganisms-10-00411-s001.zip › microorganisms-1555127-supplementary.pdf]

Table S1. Odds ratios determined from bivariable logistic regression analysis of ESBL *E. coli* prevalence by age, antibiotic administration, and treatment events.

Independent Variables	ESBL-producing *E. coli* Prevalence											
	Hutch			Weaned			Yearling			All Ages		
	OR	P-value	95% CI	OR	P-value	95% CI	OR	P-value	95% CI	OR	P-value	95% CI
Age	-	-		-	-		-	-		5.54	< 0.001[*]	3.19-9.60
Hutch (base)	-	-		0.47	0.094	0.19-1.14	0.03	< 0.001[*]	0.01-0.09	-	-	-
Weaned (base)	2.13	0.094	0.88-5.16	-	-		0.06	< 0.001[*]	0.02-0.18	-	-	-
Yearling (base)	38.68	< 0.001[*]	11.38-131.48	18.16	< 0.001[*]	5.62-58.63	-	-		-	-	-
Antibiotics Administered												
Aminoglycoside	1[om]	-	-	1[om]	-	-	1[om]	-	-	1[om]	-	-
Cephalosporin	0.58	0.431	0.15-2.27	0.82	0.811	0.16-4.15	1[em]	-	-	0.56	0.119	0.27-1.16
Florfenicol	1[em]	-	-	1.70	0.388	0.51-5.63	3.00	0.357	0.29-31.01	0.85	0.634	0.44-1.64
Fluoroquinolone	0.99	0.987	0.22-4.48	0.61	0.633	0.08-4.72	1[em]	-	-	1.34	0.526	0.54-3.33
Macrolide	1.94	0.562	0.21-18.07	0.69	0.536	0.21-2.25	4.67	0.197	0.45-48.41	0.66	0.253	0.33-1.34
Tetracycline	-	-	-	1[om]	-	-	1[om]	-	-	0.16	0.000	0.07-0.34
Tetracycline (w/o Prophylactic)	-	-	-	1[em]	-	-	1.37	0.795	0.13-14.77	0.08	0.015	0.01-0.60
Penicillin	1[om]	-	-	1[om]	-	-	1[om]	-	-	1[om]	-	-
Penicillin (w/o Prophylactic)	1.90	0.454	0.35-10.28	1.31	0.771	0.22-7.94	1[em]	-	-	1.99	0.133	0.81-4.90
Treatment Event												
Respiratory	1.33	0.685	0.33-5.35	0.88	0.852	0.22-3.52	1.45	0.755	0.14-15.15	0.75	0.407	0.39-1.47
Scours	0.65	0.536	0.17-2.55	0.82	0.811	0.16-4.15	0.40	0.438	0.04-4.10	0.55	0.098	0.27-1.12
Bloat	1.67	0.555	0.31-9.08	0.94	0.953	0.14-6.25	1[em]	-	-	1.67	0.275	0.67-4.19
Eyes	-	-	-	-	-	-	1.58	0.706	0.15-17.25	0.12	0.050	0.01-1.00

Data includes use of prophylactic tetracycline in weaned and yearling age groups, as well as penicillin and neomycin (aminoglycoside) in all ages, unless indicated as without (w/o). [om] omitted due to collinearity. [em] empty. [*] p-value significant at 0.05 alpha.

Table S2. Percentages of calves treated with antibiotics for health events requiring treatment, all 147 calves had antibiotic use data available from hutch (n = 48), weaned (n = 49), and yearling (n = 50) age groups.

Antibiotic	Hutch		Weaned		Yearling		All Ages	
Administered	Treated (%)	95% CI	Treated (%)	95% CI	Treated (%)	95% CI	Treated (%)	95% CI
Cephalosporin	35.42	22.16-554	14.29	5.94-27.24	36.00	22.92-50.81	28.57	21.43-36.60
Phenicol	10.42	3.47-22.66	65.31	50.36-78.33	52.00	37.42-66.34	42.86	34.74-51.27
Fluoroquinolone	27.08	15.28-41.85	8.16	2.27-19.60	10	3.33-21.81	14.97	9.62-21.78
Macrolide	14.58	6.07-27.76	36.73	23.42-51.71	42.00	28.19-56.79	31.29	23.91-39.45
Penicillin	27.08	15.28-41.85	12.24	4.63-24.77	10	3.33-21.81	16.33	10.75-23.31
Tetracycline	0.00	0.00-7.40	6.12	1.28-16.87	20	10.03-33.72	8.84	4.79-14.65

Data does not include antibiotics administration of prophylactic tetracycline in weaned and yearling age groups, as well as penicillin and neomycin (aminoglycoside) in all ages.

Table S3. Bivariable linear regression for age, antibiotic treatments, and log10CFU/g feces growth on MacConkey, MacConkey with 16µg/mL tetracycline, MacConkey with 32µg/mL erythromycin, MacConkey with 4µg/mL ceftriaxone, and MacConkey with 1µg/mL ciprofloxacin agars.

Independent Variables	log10CFU/g feces									
	MacConkey		MacConkey+AXO		MacConkey+TET		MacConkey+ERY		MacConkey+CIP	
Univariate Models	Coef.	P-value	Coef.	P-value	Coef.	P-value	Coef.	P-value	Coef.	P-value
Age	0.46	0.015*	2.03	< 0.001**	1.58	0.002**	0.55	0.011*	1.17	0.009**
ESBL-producing *E. coli*	0.62	0.050*	1.51	0.093	2.73	0.001**	0.85	0.018*	0.46	0.557
Antibiotics Administered										
Cephalosporin	-0.33	0.317	0.48	0.612	-1.52	0.105	-0.58	0.124	0.72	0.370
Phenicol	0.06	0.853	-1.94	0.026*	0.36	0.698	0.11	0.764	-0.47	0.549
Fluoroquinolone	-0.36	0.402	0.73	0.547	-0.32	0.797	-0.37	0.461	-0.16	0.877
Macrolide	-0.59	0.061	-1.46	0.103	-1.91	0.032*	-0.56	0.128	-0.86	0.268
Tetracycline	-0.57	0.091	-3.82	< 0.001**	-1.80	0.058	-0.67	0.083	-1.91	0.015*
Tetracycline (w/o Prophylactic)	-0.48	0.374	0.21	0.891	-1.65	0.279	-0.56	0.367	0.69	0.597
Penicillin	0.00	-	0.00	-	0.00	-	0.00	-	0.00	-
Penicillin (w/o Prophylactic)	-0.13	0.765	2.00	0.093	-0.38	0.759	-0.35	0.475	0.87	0.403
Treatment Event										
Respiratory	-0.20	0.681	-3.15	0.013*	-0.52	0.702	-0.27	0.619	-0.49	0.669
Bloat	-0.13	0.765	2.00	0.093	-0.38	0.759	-0.35	0.475	0.87	0.403
Scours	0.16	0.650	0.89	0.352	-0.26	0.791	0.14	0.724	0.96	0.241
Eyes	-0.28	0.665	-0.02	0.993	-2.10	0.252	-0.32	0.672	0.17	0.911

AXO – ceftriaxone, TET – tetracycline, ERY – erythromycin, CIP – ciprofloxacin. * p-value significant at 0.05 alpha, ** p-value significant at 0.01 alpha.

Table S4. Bivariable linear regression for age, antibiotic treatments, and difference of log10CFU/g feces growth between MacConkey and MacConkey agars supplemented with antibiotics (erythromycin, tetracycline, ciprofloxacin, or ceftriaxone at same concentration as Table S3).

Independent Variables	Difference between log10CFU/g Feces							
	MacConkey+AXO		MacConkey+TET		MacConkey+ERY		MacConkey+CIP	
Univariate Models	Coef.	P-value	Coef.	P-value	Coef.	P-value	Coef.	P-value
Age	-1.57	< 0.001**	-1.12	0.003**	-0.09	0.161	-0.71	0.059
ESBL-producing *E. coli*	-0.89	0.252	-2.11	< 0.001**	-0.22	0.031*	0.16	0.803
Antibiotics Administered								
Cephalosporin	-0.81	0.307	1.18	0.077	0.25	0.020*	-1.06	0.101
Phenicol	2.00	0.006**	-0.30	0.653	-0.05	0.631	0.53	0.404
Fluoroquinolone	-1.10	0.287	-0.05	0.959	0.00	0.984	-0.20	0.814
Macrolide	0.86	0.261	1.31	0.040*	-0.04	0.730	0.27	0.677
Tetracycline	3.25	< 0.001**	1.23	0.072	0.10	0.396	1.34	0.038*
Tetracycline (w/o Prophylactic)	-0.69	0.594	1.17	0.285	0.08	0.664	-1.16	0.266
Penicillin	0.00		0.00		0.00		0.00	
Penicillin (w/o Prophylactic)	-2.13	0.033*	0.25	0.779	0.22	0.111	-1.00	0.235
Treatment Event								
Respiratory	2.95	0.006**	0.32	0.740	0.08	0.632	0.29	0.753
Bloat	-2.13	0.033*	0.25	0.779	0.22	0.111	-1.00	0.235
Scours	-0.74	0.367	0.42	0.553	0.02	0.879	-0.80	0.227
Eyes	-0.27	0.864	1.82	0.165	0.03	0.876	-0.46	0.720

AXO – ceftriaxone, TET – tetracycline, ERY – erythromycin, CIP – ciprofloxacin. * p-value significant at 0.05 alpha, ** p-value significant at 0.01 alpha.

Table S5. Odds ratios determined from bivariable logistic regression analysis of *mph*(A) and *qnrB* prevalence in ESBL-producing *E. coli* isolates by bla_{CTX-M} variant group.

Independent Variables	Prevalence of *mph*(A) and *qnrB* in ESBL *E. coli*					
	mph(A)		*qnrB*		*mph*(A) and *qnrB*	
	OR (95% CI)	P-value	OR (95% CI)	P-value	OR (95% CI)	P-value
bla_{CTX-M} group 1	4.34 (2.01-9.38)	< 0.001**	0.24 (0.13-0.45)	< 0.001**	2.14 (0.70-6.54)	0.183
bla_{CTX-M} group 9	0.23 (0.11-0.50)	< 0.001**	4.17 (2.24-7.78)	< 0.001**	0.47 (0.15-1.43)	0.183

* p-value significant at < 0.05 alpha, ** p-value significant at < 0.01 alpha

Table S6. Whole genome sequencing data for ESBL *E. coli* isolates submitted to NCBI under BioProject number PRJNA766656.

Sample ID	BioSample Accession	Sequencing Coverage	Total Length (bp)	Avg. R1 R2 seq. Length
5-B-3-Ecoli-Feb2020-1	SAMN24694688	45.5852766	4973276	198.028488
21-B-6-Ecoli-Feb2020-2	SAMN24694689	40.4696721	5497489	179.285821
13-W-3-Ecoli-Feb2020-3	SAMN24694690	53.2780201	5051664	184.499467
47-W-3-Ecoli-Feb2020-2	SAMN24694691	36.2399923	5543127	179.682823
5-H-6-Ecoli-Feb2020-3	SAMN24694692	45.8621664	4940292	191.349298
18-H-6-Ecoli-Feb2020-2	SAMN24694693	48.8484911	4604754	184.893311
19-H-6-Ecoli-Feb2020-1	SAMN24694694	38.2499068	4681954	179.285821
34-H-6-Ecoli-Feb2020-1	SAMN24694695	49.0329741	4931317	192.349781
